# Age-Related Changes in Bimanual Instrument Playing with Rhythmic Cueing

**DOI:** 10.3389/fpsyg.2017.01569

**Published:** 2017-09-26

**Authors:** Soo Ji Kim, Sung-Rae Cho, Ga Eul Yoo

**Affiliations:** ^1^Music Therapy Education, Graduate School of Education, Ewha Womans University, Seoul, South Korea; ^2^Ewha Music Rehabilitation Center, Seoul, South Korea; ^3^Department and Research Institute of Rehabilitation Medicine, Yonsei University College of Medicine, Seoul, South Korea; ^4^Brain Korea 21 PLUS Project for Medical Science, Yonsei University College of Medicine, Seoul, South Korea; ^5^Rehabilitation Institute of Neuromuscular Disease, Yonsei University College of Medicine, Seoul, South Korea; ^6^Department of Music Therapy, Graduate School, Ewha Womans University, Seoul, South Korea

**Keywords:** older adults, cognitive aging, bimanual coordination, instrument playing, timing accuracy

## Abstract

Deficits in bimanual coordination of older adults have been demonstrated to significantly limit their functioning in daily life. As a bimanual sensorimotor task, instrument playing has great potential for motor and cognitive training in advanced age. While the process of matching a person’s repetitive movements to auditory rhythmic cueing during instrument playing was documented to involve motor and attentional control, investigation into whether the level of cognitive functioning influences the ability to rhythmically coordinate movement to an external beat in older populations is relatively limited. Therefore, the current study aimed to examine how timing accuracy during bimanual instrument playing with rhythmic cueing differed depending on the degree of participants’ cognitive aging. Twenty one young adults, 20 healthy older adults, and 17 older adults with mild dementia participated in this study. Each participant tapped an electronic drum in time to the rhythmic cueing provided using both hands simultaneously and in alternation. During bimanual instrument playing with rhythmic cueing, mean and variability of synchronization errors were measured and compared across the groups and the tempo of cueing during each type of tapping task. Correlations of such timing parameters with cognitive measures were also analyzed. The results showed that the group factor resulted in significant differences in the synchronization errors-related parameters. During bimanual tapping tasks, cognitive decline resulted in differences in synchronization errors between younger adults and older adults with mild dimentia. Also, in terms of variability of synchronization errors, younger adults showed significant differences in maintaining timing performance from older adults with and without mild dementia, which may be attributed to decreased processing time for bimanual coordination due to aging. Significant correlations were observed between variability of synchronization errors and performance of cognitive tasks involving executive control and cognitive flexibility when asked for bimanual coordination in response to external timing cues at adjusted tempi. Also, significant correlations with cognitive measures were more prevalent in variability of synchronization errors during alternative tapping compared to simultaneous tapping. The current study supports that bimanual tapping may be predictive of cognitive processing of older adults. Also, tempo and type of movement required for instrument playing both involve cognitive and motor loads at different levels, and such variables could be important factors for determining the complexity of the task and the involved task requirements for interventions using instrument playing.

## Introduction

Cognitive aging, defined as age-related changes in perceptual and cognitive performance, results in less efficient use of mental resources (Park, 2000). As such, cognitive aging is manifested by slower processing speed, decreased mental capacity during performance of more complex cognitive tasks, and inflexible attentional ability (Han, 2016). Cognitive decline due to dementia interferes with cognitive and motor functioning required for daily living activities at different levels (Giebel et al., 2014). During earlier stages of dementia, affected people demonstrate less executive control, which also impacts motor control, such as a safe gait (van der Wardt et al., 2015). The cognitive difficulties experienced in the early stages of dementia impact one’s self-confidence and can lead to anxiety, depression, and withdrawal from activities (Giebel et al., 2014; Han, 2017), which can exacerbate symptoms. As such, there have been increasing calls for interventions targeting the cognitive functioning of older adults with earlier stages of dementia, where the disease has not yet progressed to the point of severe cognitive impairment.

With increasing evidence supporting a correlation between cognitive and motor functions, research has demonstrated that cognitive decline in advanced age also affects motor coordination in association with attentional control and executive function (Temprado et al., 2001; Fujiyama et al., 2013). For example, slower speed or decreased accuracy in performing sequential finger movements in older adults was found to be associated with mental representation of such motor information, which was also supported by increased activation in non-motor areas as well as motor areas in the brain (Caçola et al., 2013). Meanwhile, level of cognitive impairment affects the response time during simple finger tapping in association with working memory capacity (Halliday et al., 2016). With regard to bimanual coordination, which requires the integration of complex neural systems (including motor and perceptual systems; Vaillancourt and Newell, 2002), older adults show decreased accuracy and stability (Lipsitz, 2004). Age-related decline specifically impacts timing processing and control of attentional load, which are viewed as primary factors in task performance of interlimb coordination in the upper extremity (Krampe et al., 2010). In addition, cognitive impairment influences coordination, such that decreased imitation ability of bimanual gestures has been observed in older adults in the early stages of dementia (Nagahama et al., 2015).

Deficits in bimanual coordination have been demonstrated to significantly limit older adults’ functioning in daily life (Kilbreath and Heard, 2005). Due to these changes, difficulty in generating appropriate responses for task execution or manipulation of objects is observed in older adults (Tucker et al., 2008). Accordingly, there is interest in examining how task performance involving bimanual coordination operates and how such motor coordination can be enhanced. When older adults perform a task involving bimanual coordination, the level of performance varies depending on the direction (iso-directional vs. opposite directional) of the involvement of each limb (Meesen et al., 2006) and the speed of movement (Carson et al., 1995; Fujiyama et al., 2013). When the limbs move in opposite directions (Fujiyama et al., 2009) or at a faster speed, the performance level of older adults decreases (Fujiyama et al., 2010). Given these findings concerning the factors that facilitate or limit bimanual coordination of older adults such as the type of movement and tempo, further research is needed to develop evidence-based training to facilitate such ability to control timed movements in older populations (Voelcker-Rehage et al., 2011).

A common task involving bimanual coordination in music-related experiences is instrument playing. Instrument playing necessitates movement of the upper limbs and grasping movements, which require interlimb temporal coordination. It involves both symmetric and non-symmetric coordination depending on the type of instrument. In other words, the particular playing methods associated with different instruments facilitate specific temporal timing and movement demands. Given that deficits in bimanual coordination in older populations are attributed to the reduced size of the corpus callosum, which may interfere with interhemisphreic interaction (Sullivan and Pfefferbaum, 2002; Bartzokis et al., 2004; Head et al., 2004; Fling and Seidler, 2012), instrument playing involving upper limb movement using both hands may be promising in recruiting and optimizing neural networks in both the right and left hemispheres of older adults. Engagement in instrument playing in older adults was found to relate to reduced occurrence of dementia within twins (Balbag et al., 2014). Intensive engagement in bimanual synchronized finger movement via piano playing led to more efficient recruitment of motor networks in the brain (Schlaug, 2001) and a higher level of regularity in synchronization to auditory cues and decreased reaction time to such cues (Haslinger et al., 2004). As such, playing instruments with auditory temporal coupling requires motor and cognitive control, which accordingly mediates cognitive stimulation and accordingly predicts preserved cognitive functioning in terms of visual-spatial and executive processing (Hanna-Pladdy and Gajewski, 2012).

As a bimanual sensorimotor task, instrument playing has potential for motor and cognitive training with older adults, with evidence of beneficial effects for cognitive enhancement, gait function and fall risks (Trombetti et al., 2011). Individualized piano instruction that involves multimodal sensorimotor integration and temporal and spatial processing was found to effectively facilitate perceptual processing in older adults, which indicates the potential benefits of musical instruction for cognitive aging (Bugos et al., 2007). Intervention with the dual task of handling percussion instruments while walking effectively influences gait parameters and dual task performance, which eventually reduces fall risks. Performance of musical tasks, including rhythm playing, was documented to contribute to delayed cognitive decline in older adults with mild to moderate dementia (Chu et al., 2014).

When performing tasks involving motor control, such as playing a musical instrument, the provision of auditory rhythmic cueing can function as an effective agent for intervening in sensorimotor processing and the timed control of movements (Chen et al., 2006; LaGasse and Knight, 2011). The process of matching a person’s repetitive movements to auditory rhythmic cueing was documented to facilitate the precise execution of sequenced motor tasks and increase efficiency in brain activation engaged in such motor control (Witt et al., 2008). Also, the use of repetitive rhythmic cueing presents precise timing information and thereby enhances predictive processing, which leads to decreases in attentional demand and increases in efficiency of motor and attentional control when continuously maintaining rhythmic movements (Ackerley et al., 2011; LaGasse and Knight, 2011; Pecenka et al., 2013). Its application to motor rehabilitation also indicates that rhythmic cueing is an effective agent when intervening with gait of individuals who benefit from external cueing for motor coordination. Previous research demonstrates that older adults in the later stages of dementia could match their gait to external auditory cueing with decreased physical support compared to walking without rhythmic cueing (Clair and O’Konski, 2006). Despite evidence of increased temporal coordination when matched to external cueing in terms of motor and cognitive performance, investigation into whether the synchronization process involving bimanual coordination impacts cognitive and motor functioning in older populations is relatively limited. Therefore, the current study aimed to examine how task performance of bimanual instrument playing with rhythmic cueing differed depending on cognitive aging. The level of performance of bimanual tapping tasks (i.e., synchronization errors) was analyzed and compared depending on the type of tasks (using both hands simultaneously and in alternation) and the tempo of cueing. It also investigated whether there were differences in such performance among young adults and older adults with and without mild dementia. Such results will contribute to better understanding of the factors associated with bimanual coordination when constructing a task for intervening in cognitive aging.

## Materials and Methods

### Participants

All procedures and ethical issues related to this study were reviewed and approved by the Institutional Review Board of Ewha Womans University (IRB No. 89-7). Young adults aged 20–40 and older adults aged 65 and over were initially recruited from universities, local community centers, and centers for older adults with dementia: 23 young adults (YA), 22 healthy older adults (OA), and 17 older adults with mild dementia (MD). Eight males and 15 females were recruited for the YA group; 5 males and 17 females were recruited for the OA group; and 6 males and 11 females were recruited for the MD group. An informed consent was obtained from each participant prior to the study. The Korean versions of the Mini-Mental State Examination (MMSE) and Geriatric Depression Scale (GDS) were used to screen for the inclusion criteria for participants. Healthy YA and OA individuals were included if they obtained a score of 24 or higher on the MMSE. Older adults with mild dementia who had been diagnosed with Alzheimer’s disease or vascular dementia were recruited and screened for MMSE scores between 19 and 23. Two young adults scored over 14 on the GDS, and in the OA group without diagnosis of dementia, two participants obtained less than 19 on the MMSE. They were excluded from further data analysis. The remaining participants indicated no discernible hearing deficits on a completion of a 10-item questionnaire on hearing loss in various listening conditions. They were also able to follow verbal commands and perform visuospatial tasks, such as writing. In the final analysis, a total of 58 participants were included. Demographic information is displayed in **Table 1**.

**Table 1:** Demographic information of participants.

Parameter	YA (*n* = 21)	OA (*n* = 20)	MD (*n* = 17)
Gender (M:F)	8:13	5:15	6:11
Age, years (*M* ±*SD*)	28.3 ± 6.6	75.8 ± 8.2	77.6 ± 3.7
MMSE (*M* ±*SD*)	29.5 ± 0.6	27.3 ± 1.7	21.1 ± 1.5
GDS (*M* ±*SD*)	5.1 ± 3.4	7.4 ± 6.2	7.0 ± 4.1
Dominant hand (Rt:Lt)	21:0	20:0	17:0

*MMSE, Mini-Mental State Examination; GDS, Geriatric Depression Scale.*

### Stimuli

A 12-inch electronic drum pad (Alesis PercPad, Cumberland, RI, United States) was used with drum sticks. A musical instrument digital interface (MIDI) was used to transfer the signals obtained during drum tapping to the Cubase 5 (Steinberg Media Technologies AG, Hamburg, Germany). The MIDI-generated signals enabled the collection and analysis of data on the timing of each tapping measured as a unit of seconds. For the provision of rhythmic cueing, a MIDI software-embedded metronome was used.

### Measurement

For cognitive measurements, the Digit Span Test (DST) and Trail Making Test (TMT) were used. The DST (Kaplan et al., 1991; Kang et al., 2002) is used to assess working memory and consists of two subtests: Digit Span Forward (DSF), the test of ability to recall three- to nine-digit numbers in a presented order, and Digit Span Backward (DSB), the test of ability to recall a series of numbers in a reverse order than initially presented (Jahanshahi et al., 2009). The Korean version of TMT (Reitan, 1956; Yi et al., 2007) consists of two subtests (TMT-A and TMT-B) and measures working memory and executive function. During the TMT-A, a participant draws a line connecting circled numbers in ascending order from 1 to 15. The TMT-B requires the participant to draw a line connecting circled numbers and words (i.e., the words for the days of the week) alternatively as in 1, Monday, 2, Tuesday, and so on. While TMT-A and TMT-B both measure visuospatial attention, sequencing, speed of processing and working memory, executive functioning, and cognitive flexibility, the TMT-B also requires attentional control and inhibition and set-shifting task performance (Arbuthnott and Frank, 2000).

For drum tapping tasks, each participant was initially instructed to tap an electronic drum using both hands at their preferred tempo while tapping with both hands simultaneously and in alteration. Then they tapped the drum in time to the rhythmic cueing provided. Such drum tapping tasks were also implemented in two conditions: simultaneous and alternative tapping tasks. When they performed each drum tapping task, they were instructed to maintain the task until they were said to stop. Prior to each trial, participants were presented with a practice trial and after confirming that they understood the task, each trial began. They maintained at least 30 taps for each trial and the duration of its trial was 20–30 s. Regarding the provision of rhythmic cueing, the tempo of cueing was adjusted according to five conditions: each participant’s preferred tempo measured during self-paced tempo and adjusted tempo at ±10 and ±20% of the baseline tempo. The order of presenting tasks was randomly determined for each participant prior to the test. The drum tapping tasks provided to each participant are displayed in **Table 2**.

**Table 2:** Drum tapping tasks.

Task	Involvement of limbs	Provision of rhythmic cueing	Tempo of rhythmic cueing
Self-paced tapping	Simultaneous	N	NA
	Alternative	N	NA
Tapping to rhythmic cueing	Simultaneous	Y	Baseline /±10% /±20%
	Alternative	Y	Baseline /±10% /±20%

*Y, yes; N, no; NA, not applicable; +, faster; -, slower.*

### Procedures

The current study was individually conducted in a quiet room of a university, a local community center, and a center for older adults with dementia where participants were recruited. In order to minimize the noise in the environment to an equivalent level across settings, isolated places without adjacent rooms where noise could be produced were selected. For each participant who agreed to participate in this study, cognitive measures and drum tapping tasks were administered. During drum tapping tasks, each participant was instructed to tap the drum at their preferred tempo (self-paced tempo) and at a tempo that matched the presented cueing (tapping to rhythmic cueing tasks).

### Data Collection and Analysis

**FIGURE 1:**
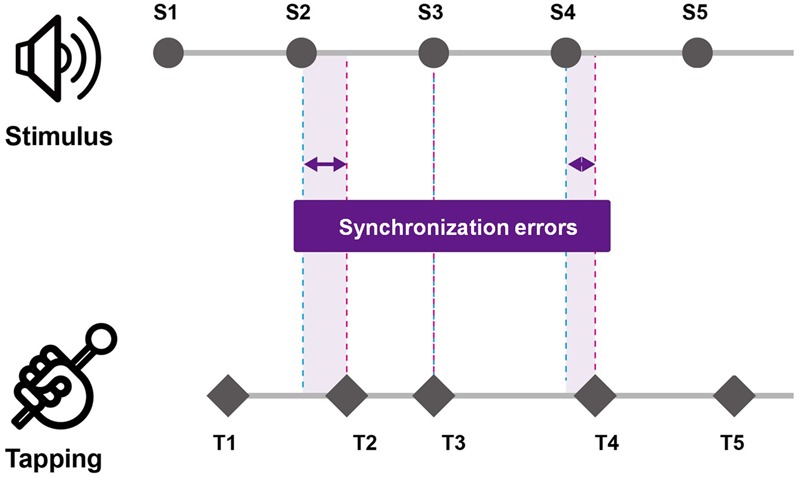
Measurement of synchronization errors of tapping task performance. Synchronization errors were measured by calculating the difference in the timing of the tap and the onset of rhythmic cueing.

The DST was scored with the number of items that each participant accurately recalled in the presented order (DSF) and the reverse order (DSB). For the TMT, both the TMT-A and TMT-B were scored with the time to complete the test. During self-paced tapping, the intervals in the timing of each two consecutive taps were calculated [i.e., inter-tap interval (ITI)] and a total of 29 ITIs for each trial were averaged. Also, in order to measure the regularity of tapping, the variability (standard deviation) of ITI was calculated. And for drum tapping task with rhythmic cueing, the synchronization errors were measured via two parameters: mean synchronization errors and variability of synchronization errors. First, synchronization errors were measured by calculating the difference in the timing of the tap and the onset of rhythmic cueing (see **Figure 1**). For the mean synchronization errors, the absolute value of each difference in the timing of the tap and the onset of rhythmic cueing was calculated and averaged for each trial. Then variability of synchronization errors was measured with the average coefficient of variation by calculating the standard deviation of the collected time differences divided by the mean inter-stimulus interval of the rhythmic cueing. For each type of tapping task (i.e., simultaneous and alternative tapping), the measures of synchronization errors were analyzed and compared across the age group by conducting a mixed between-within subjects ANOVA with the group as a between-group factor and with tempo of the cueing as within-group factor. For *post hoc* analyses, the Bonferroni correction was used. Furthermore, Spearman’s correlations were conducted to examine the relationship between cognitive measures and synchronization errors-related parameters for each playing task condition (i.e., simultaneous and alternative tapping conditions).

## Results

This study investigated whether the performance of bimanual tapping to rhythmic cueing differed depending on the participants’ cognitive aging in terms of synchronization errors.

### Cognitive Measures of Participants

**FIGURE 2:**
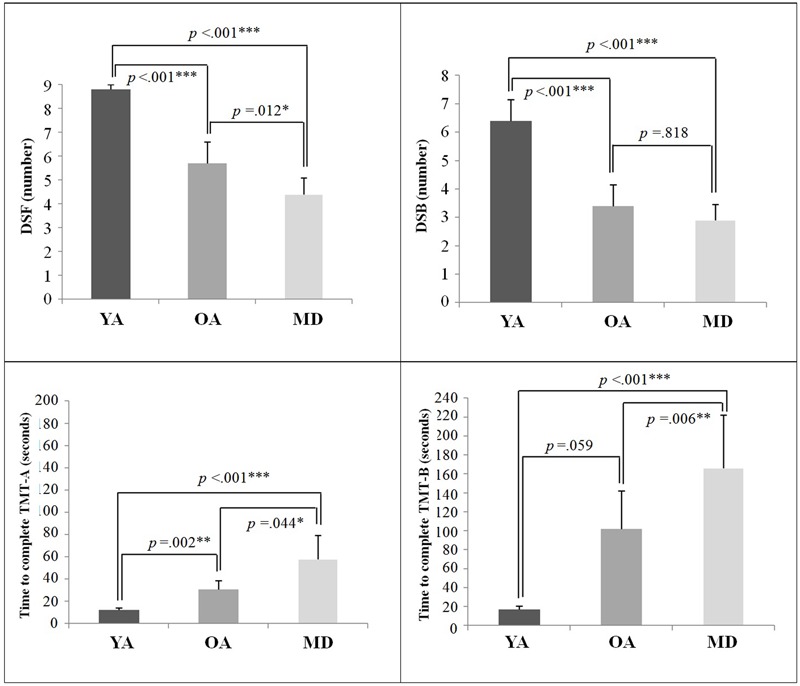
Group comparison of cognitive measures.

The descriptive results of the cognitive measures of the DST and TMT tests are displayed in **Table 3**. A one-way ANOVA showed that there were significant group differences on all cognitive measures. *Post hoc* analyses with the Bonferroni correction showed that for DSF and TMT-A measures, the YA group demonstrated significantly greater performance than the OA and MD groups. The OA group also recalled significantly more digits, compared to the MD group. For DSB, while the YA group recalled significantly longer digits than the OA and MD groups, the comparison between the OA and MD groups did not reach statistical significance. For the TMT-B measure, the YA and OA groups completed the test within significantly less time, compared to the MD group. There was no significant difference in the time to complete the TMT-B between the YA and OA groups. Results on the group comparisons in terms of cognitive measures are displayed in **Figure 2**.

**Table 3:** Results of cognitive measures for each group.

Parameter	YA (*n* = 21)	OA (*n* = 20)	MD (*n* = 17)	*F*(2,55)	*P*
DST					
DSF (*M* ±*SD*)	8.8 ± 0.4	5.7 ± 1.8	4.4 ± 1.4	57.085	<0.001^∗∗∗^
DSB (*M* ±*SD*)	6.4 ± 1.5	3.4 ± 1.5	2.9 ± 1.1	35.995	<0.001^∗∗∗^
TMT					
TMT-A, sec (*M* ±*SD*)	12.3 ± 4.0	30.8 ± 15.7	57.7 ± 43.0	15.454	<0.001^∗∗∗^
TMT-B, sec (*M* ±*SD*)	17.0 ± 6.8	101.9 ± 80.6	166.2 ± 112.0	17.915	<0.001^∗∗∗^

*DST, Digit Span Test; DSF, Digit Span Forward; DSB, Digit Span Backward; TMT, Trail Making Test. *^∗∗∗^p* < 0.001.*

### Self-Paced Bimanual Tapping

In this study, each participant was instructed to tap the electronic drum with both hands simultaneously and then alternatively using one hand and then the other at their preferred tempo. The results of a one-way ANOVA showed that there were significant differences in ITI among the groups during both simultaneous tapping and alternative tapping (see **Table 4**). *Post hoc* analyses with the Bonferroni correction showed that the YA group exhibited significantly slower tapping than the OA group during simultaneous (*p* = 0.005) and alternative bimanual tapping (*p* = 0.005). Paired comparisons between the YA and MD groups (*p* = 0.062 for simultaneous tapping and *p* = 1.000 for alternative tapping) and between the OA and MD groups (*p* = 0.092 for simultaneous tapping and *p* = 1.000 for alternative tapping) did not reach statistical significance.

**Table 4:** Mean ITI (seconds) during self-paced tapping.

	Mean ITI (*M* ± *SD*)				
Type of tapping	YA (*n* = 21)	OA (*n* = 20)	MD (*n* = 17)	*F*(2,55)	*p*
Simultaneous	0.606 ± 0.214	0.444 ± 0.106	0.482 ± 0.126	5.833	0.005^∗∗^
Alternative	0.519 ± 0.223	0.343 ± 0.147	0.396 ± 0.107	5.780	0.005^∗∗^

*ITI, inter-tap interval. ^∗∗^*p* < 0.01.*

With regard to tapping variability, a one-way ANOVA was conducted to see whether the level of maintaining the regularity of self-paced tapping differed depending on the group. The results showed that there were no significant differences between groups during both simultaneous tapping and alternative tapping (see **Table 5**).

**Table 5:** Tapping variability during self-paced tapping.

	Tapping variability (*M* ± *SD*)				
Type of tapping	YA (*n* = 21)	OA (*n* = 20)	MD (*n* = 17)	*F*(2,55)	*p*
Simultaneous	0.024 ± 0.012	0.030 ± 0.028	0.028 ± 0.020	0.395	0.675
Alternative	0.032 ± 0.017	0.049 ± 0.059	0.038 ± 0.023	0.993	0.400

### Mean Synchronization Errors during Bimanual Tapping

**FIGURE 3:**
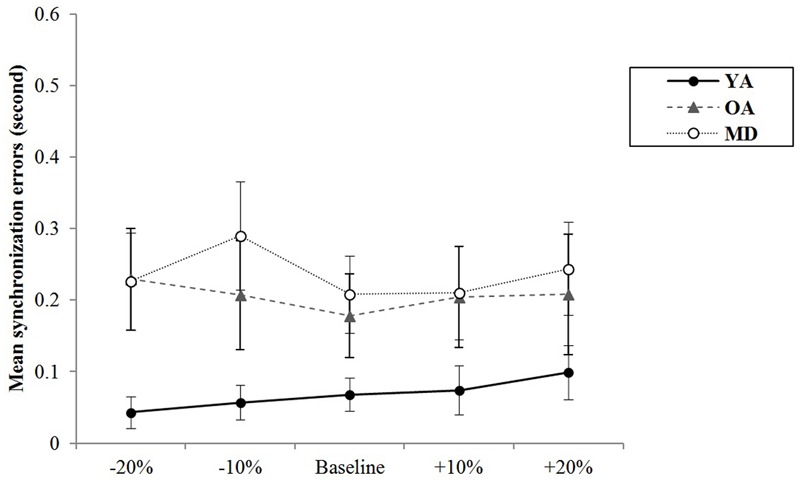
Group comparison of mean synchronization errors during simultaneous bimanual tapping.

In terms of measures of synchronization errors, in order to investigate the degree of synchronization errors without consideration of the tendency to tap (i.e., the tendency to tap before vs. after the provision of cueing), the absolute values of synchronization errors were calculated and compared across groups. Increases in such value represent increased synchronization errors, indicating lower timing accuracy. Conversely, decreased values indicate higher timing accuracy. For simultaneous tapping, the results of a mixed model repeated measures ANOVA showed that the main effect of tempo was statistically significant, *F*(4,220) = 3.657, *p* = 0.007, η^2^ = 0.062, Power = 936. A *post hoc* analysis with a Bonferroni correction demonstrated that the +10% tempo condition elicit significantly less synchronization errors than the -20% tempo condition (*p* = 0.020). The other paired comparisons did not reach statistical significance. The interaction effect between the tempo and group was not significant, *F*(8,220) = 1.702, *p* = 0.099, η^2^ = 0.058, Power = 769, indicating that changes in synchronization errors depending on the tempo condition were similar across the group. The group significantly affected the mean synchronization errors, *F*(2,55) = 5.065, *p* = 0.010, η^2^ = 0.156, Power = 943. A *post hoc* analysis with a Bonferroni correction demonstrated that the YA group showed significantly less synchronization errors than the MD group (*p* = 0.009). The other paired comparisons did not reach statistical significance (see **Table 6** and **Figure 3**).

**Table 6:** Mean synchronization errors during bimanual tapping depending on the tempo condition.

Tapping task	Group	Synchronization errors, ms (*M* ± *SD*)				
		-20%	-10%	Baseline	+10%	+20%
Simultaneous	YA (*n* = 21)	0.090 ± 0.077	0.092 ± 0.072	0.087 ± 0.075	0.088 ± 0.072	0.083 ± 0.067
	OA (*n* = 20)	0.131 ± 0.070	0.103 ± 0.063	0.087± 0.055	0.094 ± 0.062	0.101 ± 0.061
	MD (*n* = 17)	0.165 ± 0.078	0.148 ± 0.073	0.158 ± 0.088	0.109 ± 0.047	0.144 ± 0.060
Alternative	YA (*n* = 21)	0.080 ± 0.062	0.087 ± 0.063	0.087± 0.079	0.068 ± 0.067	0.074 ± 0.081
	OA (*n* = 20)	0.099 ± 0.060	0.099 ± 0.072	0.086 ± 0.031	0.070 ± 0.032	0.084 ± 0.040
	MD (*n* = 17)	0.169 ± 0.112	0.149 ± 0.054	0.131 ± 0.047	0.147 ± 0.056	0.181 ± 0.085

*YA: young adults; OA: older adults; MD: mild dementia.*

**FIGURE 4:**
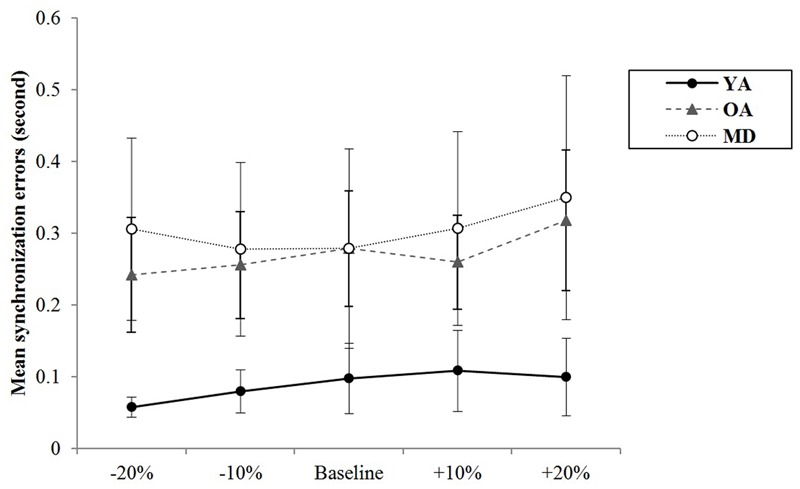
Group comparison of mean synchronization errors during alternative bimanual tapping.

For alternative tapping, the results of a mixed model repeated measures ANOVA showed that the main effect of tempo was not statistically significant, *F*(4,220) = 2.278, *p* = 0.082, η^2^ = 0.040, Power = 764, and the interaction effect between tempo and group was also not significant, *F*(8,220) = 1.688, *p* = 0.102, η^2^ = 0.058, Power = 761, indicating that changes in synchronization errors depending on the tempo condition were similar across the groups. The group significantly affects the mean synchronization errors, *F*(2,55) = 11.503, *p* < 0.001, η^2^ = 0.295, Power = 0.999. A *post hoc* analysis with a Bonferroni correction demonstrated that the YA and OA groups showed significantly less synchronization errors than the MD group (*p* < 0.001 for the YA-MD comparison and *p* = 0.001 for the OA-MD comparison). The other paired comparisons did not reach statistical significance (see **Table 6** and **Figure 4**).

### Variability of Synchronization Errors during Bimanual Tapping

For the variability of synchronization errors, the descriptive results of the coefficient of variation during the simultaneous tapping condition are displayed in **Table 7**. The YA group showed the lowest values for variability of synchronization errors in all tempo conditions. Meanwhile, the MD group showed the highest variability of synchronization errors in all tempo conditions, except the -20% tempo condition. The OA group showed the highest variability of synchronization at -20% tempo condition and the MD showed it at -10% tempo. While the YA group showed the tendency that the variability in maintaining synchronization accuracy slightly increases as the tapping tempo gets faster, the OA and MD groups tended to show increased variability of synchronization errors at slower and faster tempi, compared to the preferred tempo.

**Table 7:** Coefficient of variation as measures for variability of synchronization errors during bimanual tapping depending on the tempo condition.

Tapping task	Group	Coefficient of variability (*M* ± *SD*)				
		-20%	-10%	Baseline	+10%	+20%
Simultaneous	YA (*n* = 21)	0.043 ± 0.044	0.057 ± 0.048	0.068 ± 0.046	0.074 ± 0.069	0.099 ± 0.076
	OA (*n* = 20)	0.229 ± 0.136	0.207 ± 0.152	0.178 ± 0.108	0.204 ± 0.131	0.208 ± 0.130
	MD (*n* = 17)	0.226 ± 0.143	0.290 ± 0.152	0.208 ± 0.117	0.210 ± 0.141	0.244 ± 0.169
Alternative	YA (*n* = 21)	0.058 ± 0.028	0.080 ± 0.060	0.098 ± 0.097	0.109 ± 0.113	0.100 ± 0.108
	OA (*n* = 20)	0.242 ± 0.127	0.256 ± 0.121	0.279 ± 0.139	0.260 ± 0.135	0.318 ± 0.170
	MD (*n* = 17)	0.306 ± 0.160	0.278 ± 0.149	0.279 ± 0.161	0.307 ± 0.131	0.350 ± 0.196

*YA: young adults; OA: older adults; MD: mild dementia.*

**FIGURE 5:**
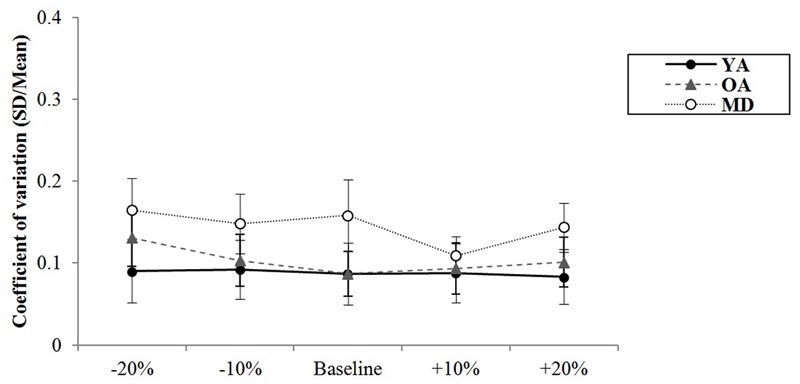
Group comparison of variability of synchronization errors during simultaneous bimanual tapping.

A mixed model of repeated measures ANOVA was conducted to see whether there were differences in variability of synchronization errors depending on the tempo condition across the groups during simultaneous tapping tasks. There was no significant main effect of the tempo, *F*(4,220) = 1.994, *p* = 0.098, η^2^ = 0.035, Power = 0.932. There was no significant interaction effect between tempo and group, *F*(8,220) = 1.785, *p* = 0.081, η^2^ = 0.061, Power = 851, indicating that the groups showed similar trends in terms of changes in the variability of synchronization errors depending on the tempo condition (see **Figure 5**). The main effect of group was statistically significant, *F*(2,55) = 16.979, *p* < 0.001, η^2^ = 0.382, Power = 0.999. *Post hoc* analyses with the Bonferroni correction demonstrated that the YA group showed significantly less variability in the synchronization errors than the OA (*p* < 0.001) and MD groups (*p* < 0.001). Comparison between the OA and MD groups did not reach statistical significance (*p* = 0.979).

During alternative bimanual tapping, the results for variability of synchronization errors for each group are displayed in **Table 7**. The YA group showed the least variability of synchronization errors in all tempo conditions. Meanwhile, the MD group showed the highest variability of synchronization errors in all tempo conditions. The YA group showed relatively fewer changes in their variability of synchronization errors in different tempo conditions. The OA group tended to show increased variability of synchronization errors at the slowest tempo. The MD group tended to show increased variability of synchronization errors at adjusted tempi.

**FIGURE 6:**
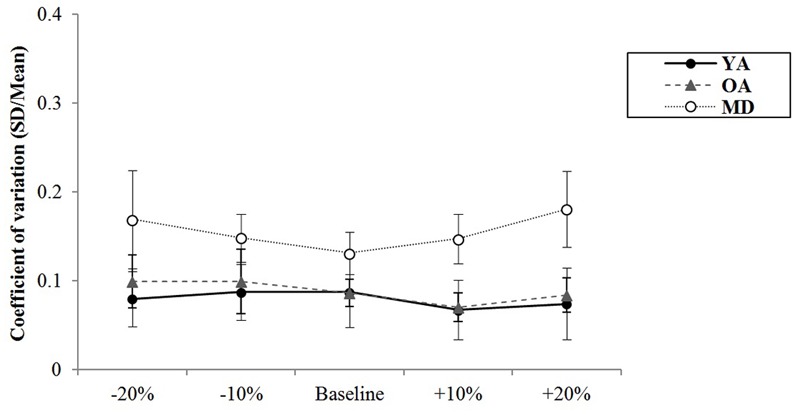
Group comparison of variability of synchronization errors during alternative bimanual tapping.

The results of a mixed model of repeated measures ANOVA showed that the main effect of the tempo was statistically significant, *F*(4,220) = 2.693, *p* = 0.039, η^2^ = 0.047, Power = 839. *Post hoc* analysis with the Bonferroni correction showed while comparison between +20% and -20% tempo condition (*p* = 0.123) and between +20% and -10% tempo condition (*p* = 0.151) elicited the greatest differences, none of the paired comparisons reached statistical significance. There were no significant interaction effects between tempo and group, *F*(8,220) = 0.704, *p* = 0.688, η^2^ = 0.025, Power = 393, indicating that there were similar trends in terms of changes in synchronization errors depending on the tempo condition across the groups (see **Figure 6**). The main effect of group was statistically significant, *F*(2,55) = 29.557, *p* < 0.001, η^2^ = 0.518, Power = 1.000. *Post hoc* analyses with a Bonferroni correction demonstrated that the YA group showed significantly less variability of the synchronization errors than the OA (*p* < 0.001) and MD groups (*p* < 0.001). Comparison between the OA and MD group did not reach statistical significance (*p* = 0.882).

### Correlation between Cognitive Measures and Timing Parameters

Finally, a Spearman’s correlation between cognitive measures and variability of synchronization errors during each of the simultaneous and alternative tapping tasks was analyzed. The correlation coefficients are displayed in **Table 8**. Correlations between cognitive measures and variability of synchronization errors ranged 0.42 to 80 and all reached statistical significance. The higher correlations over 0.70, during simultaneous tapping-related parameters indicate a significantly negative correlation between synchronization errors in -20 and -10% tempo conditions with the measure of DSF, indicating that as the difference between timing of tapping and rhythmic cueing became stable (decreased variability of synchronization), the number of digits recalled increased. During alternative tapping at the slowest tempo (-20% tempo condition), the variability of synchronization errors was highly correlated with all cognitive measures and such error at -10% tempo condition was highly correlated with DSF.

**Table 8:** Correlation between cognitive measures and variability of synchronization errors during bimanual tapping.

Variability of synchronization errors/Cognitive measures	DSF *r* (*p*)	DSB *r* (*p*)	TMT-A *r* (*p*)	TMT-B *r* (*p*)
Simultaneous tapping				
-20% tempo	-**0.71^∗∗∗^ (<0.001)**	-0.56^∗∗∗^ (<0.001)	0.59^∗∗∗^ (<0.001)	0.67^∗∗∗^ (<0.001)
-10% tempo	-**0.74^∗∗∗^ (<0.001)**	-0.60^∗∗∗^ (<0.001)	0.59^∗∗∗^ (<0.001)	0.65^∗∗∗^ (<0.001)
Baseline	-0.60^∗∗∗^ (<0.001)	-0.63^∗∗∗^ (<0.001)	0.50^∗∗∗^ (<0.001)	0.61^∗∗∗^ (<0.001)
+10% tempo	-0.60^∗∗∗^ (<0.001)	-0.55^∗∗∗^ (<0.001)	0.48^∗∗∗^ (<0.001)	0.56^∗∗∗^ (<0.004)
+20% tempo	-0.55^∗∗∗^ (<0.001)	-0.42^∗∗^ (0.001)	0.39^∗∗^ (0.002)	0.49^∗∗∗^ (<0.001)
Alternative tapping				
-20% tempo	-**0.80^∗∗∗^ (<0.001)**	-**0.73^∗∗∗^ (<0.001)**	**0.70^∗∗∗^ (<0.001)**	**0.74^∗∗∗^ (<0.001)**
-10% tempo	-**0.70^∗∗∗^ (<0.001)**	-0.68^∗∗∗^ (<0.001)	0.58^∗∗∗^ (<0.001)	0.65^∗∗∗^ (<0.001)
Baseline	-0.55^∗∗∗^ (<0.001)	-0.47^∗∗∗^ (<0.001)	0.48^∗∗∗^ (<0.001)	0.57^∗∗∗^ (<0.001)
+10% tempo	-0.63^∗∗∗^ (<0.001)	-0.63^∗∗∗^ (<0.001)	0.58^∗∗∗^ (< 0.001)	0.65^∗∗∗^ (< 0.001)
+20% tempo	-0.56^∗∗∗^ (<0.001)	-0.55^∗∗^ (0.002)	0.50^∗∗∗^ (<0.001)	0.59^∗∗∗^ (<0.001)

*Dark gray and bolded values indicates the case with correlation coefficient over 0.70 and light gray indicates the case with correlation coefficient between 0.60 and 0.70. ^∗∗^*p* < 0.01, *^∗∗∗^p* < 0.001.*

## Discussion

This study investigated whether the measures of synchronization errors during bimanual tapping tasks with rhythmic cueing differed depending on the participants’ level of cognitive aging. When asked to tap the drum at a self-paced tempo, the YA group tended to tap the drum significantly slower than the OA and MD groups; meanwhile, the tapping speed was slower for the MD group than the OA group.

Faster tapping speed observed in older adults groups may seem inconsistent with previous research showing significantly slower tapping for older adults than younger adults (Vanneste et al., 2001). However, this study may be associated with other findings showing that reaction time significantly decreased with aging, but that movement time was maintained at a similar level to younger adults (Kauranen and Vanharanta, 1996). In addition, fine motor skills have repeatedly been documented to decrease in older adults, but age-related changes in gross motor skills have not been conclusively demonstrated (Voelcker-Rehage, 2008). Given that the drum tapping task with drum mallets involves gross motor skills more than a simple finger tapping task, more controlled analysis is needed focusing on the type of involved movement. Furthermore, further studies with increased sample size and both females and males would be needed to corroborate age-related changes in and gender effects on timing measures during bimanual instrument playing. In addition, previous findings were primarily based on participants in Western countries, thereby suggesting that cultural factors, which may influence physical conditions and exposure to and engagement in musical activities, need to be considered in future studies.

The level of tapping variability was not different across the groups, indicating that the participants could maintain their regular tapping. This was consistent during both simultaneous and alternative tapping. These results indicate that the ability to perceive timing is relatively intact despite cognitive aging.

This finding supports other research demonstrating that internal timing, measured with continuous finger or hand tapping, does not elicit age-related differences (Vanneste et al., 2001; Trugeon et al., 2011). In previous studies, older adults with mild Alzheimer’s disease showed no significant differences compared to age-matched older adults and young adults when required to judge the interval of timing and discriminate different intervals (Caselli et al., 2009). These results indicate that the internal timekeeping system operates sufficiently in older adults and older adults with cognitive decline. Although research remains to be done, some brain imaging studies have explained that while decreased volume in the cerebellum and connectivity between motor-related networks negatively affect motor performance of older adults, the compensatory neural system still facilitates the maintenance of sensorimotor functioning of this population, including strengthened connectivity between the motor cortex, putamen, and cerebellum (Seidler et al., 2010). Such findings indicate that the use of external timing cues may be effectively applied to interventions for sensorimotor functioning and motor coordination for daily life activities and tasks.

With regard to synchronization errors, which measure how accurately the participants matched the rhythmic cueing, the group factor (i.e., the level of cognitive aging) resulted in significant differences during both simultaneous and alternative tapping tasks. Interesting to note was while the OA group showed less synchronization errors than the MD group, the comparison between the YA and OA groups did not reach statistical significance. These results indicate that decreases in synchronization errors may be attributed to cognitive impairment, not exclusively to aging. Although older adults could maintain their tapping, the immediacy of adjustment and motor control decreased with cognitive aging. These results also support that decreases in motor response time as reported in the literature result in slower processing speed and efficiency of performance during bimanual coordination (Trapp et al., 2012; Shetty et al., 2014). Despite relatively intact timing perception, the process of transferring perceived information into motor output may be delayed and the efficiency of coordination compromised.

In terms of variability of synchronization errors, the group factor also significantly affected such measures during both simultaneous and alternative tapping tasks. Compared to mean synchronization errors which are indicative of magnitude of asynchrony, the variability of synchronization errors (measured by coefficient of variation) indicates the synchronization precision in terms of how stable timing performance is maintained in relation to the ongoing temporal events. The YA group showed less variability in changes in synchronization errors across the tempo of cueing. Meanwhile, older adults with and without mild dementia showed increased synchronization errors at adjusted tempi. When comparing the two older adults groups, the MD group tended to show greater synchronization errors and increased variability of changes in such errors depending on the tapping task and the tempo or cueing compared to the OA group. *Post hoc* analyses demonstrated that the YA group showed significantly less variability in synchronization errors than both the OA and MD groups, indicating such group differences may be attributed to aging.

In older adults groups, greater synchronization errors at adjusted tempi than at baseline tempo. This tendency led to significant differences in the variability of synchronization errors depending on the tempo condition. Previous studies demonstrated that older adults tended to have greater difficulty in moving two limbs in opposite directions as required during alternative tapping than in moving two limbs in the same direction as required during simultaneous tapping (Mattay et al., 2002). Such findings indicate that control of two limbs separately in the opposite direction requires inhibitory control, which suppresses conflicting motor output to perform a task (Serrien et al., 2000). The results of this study suggest that decreased processing time for bimanual coordination affects the differences in timing accuracy during simultaneous tapping between younger and older adults regardless of cognitive impairment. Meanwhile, alternative tapping, in which the tempo factor significantly affect timing performance, may increase cognitive demands in addition to physical demands.

Furthermore, significantly high correlations between cognitive measures and synchronization errors during bimanual tapping indicate that bimanual coordination in response to external timing cues at adjusted tempi is involved in cognitive processing and cognitive flexibility. Temporal control with bimanual engagement was documented to require working memory and executive control (Bangert et al., 2010). In this study, alternative tapping at slower tempo was associated with a subtest of DST and TMT that require inhibitory control more than simultaneous tapping. It is also noteworthy that significant correlations with cognitive measures were observed in timing measures at adjusted tempi (slower). Such results indicate that the type of involvement of the limbs (simultaneous vs. alternative) and the tempo mediate the processing of external timing cues and motor control based on the perceived input at different levels (Ridderikhoff et al., 2008). Previous studies support that during synchronization tasks, the task to match an individual’s tapping to adjusted tempo, that is faster or slower than an individual’s preferred tempo, increases dynamic control of attentions by competing with initial expectancy mediated by internal timing and adapting to newly generated expectancy by external cueing (McAuley et al., 2006; Bangert and Balota, 2012).

In sum, the current study supports the notion that bimanual tapping may be predictive of cognitive processing of older adults. It proposes that such instrument playing can be effectively incorporated into the process of assessing and intervening in cognitive and motor functioning of older adults who show limited performance of other types of tasks due to cognitive decline. Furthermore, the current study presents implications for how instrument playing can be used as bimanual coordination tasks for older adults with varying levels of cognitive aging. Results on differences in the timing accuracy depending on the tempo and the type of task indicate that different tapping conditions require cognitive and motor loads at different levels and such variables could be an important factor for determining the complexity of the task and the involved task requirements.

## Author Contributions

SJK, S-RC, and GEY contributed to study conception and design, data acquisition and analysis, and manuscript writing.

## Conflict of Interest Statement

The authors declare that the research was conducted in the absence of any commercial or financial relationships that could be construed as a potential conflict of interest.

## References

[B1] Ackerley S. J. Stinear C. M. Byblow W. D. (2011). Promoting use-dependent plasticity with externally-paced training. *Clin. Neurophysiol.* 122 2462–2468. 10.1016/j.clinph.2011.05.011 21664864

[B2] Arbuthnott K. Frank J. (2000). Trail making test, part B as a measure of executive control: validation using a set-switching paradigm. *J. Clin. Exp. Neuropsychol.* 22 518–528. 10923061 10.1076/1380-3395(200008)22:4;1-0;FT518

[B3] Balbag M. A. Pedersen N. L. Gatz M. (2014). Playing a musical instrument as a protective factor against dementia and cognitive impairment: a population-based twin study. *Int. J. Alzheimers Dis.* 2014:836748 10.1155/2014/836748 PMC4269311 25544932

[B4] Bangert A. S. Balota D. A. (2012). Keep up the pace: declines in simple repetitive timing differentiate healthy aging from the earliest stages of Alzheimer’s disease. *J. Int. Neuropsychol. Soc.* 18 1052–1063. 10.1017/S1355617712000860 22929329 PMC3505757

[B5] Bangert A. S. Reuter-Lorenz P. A. Walsh C. M. Schachter A. B. Seidler R. D. (2010). Bimanual coordination and aging: neurobehavioral implications. *Neuropsychologia* 48 1165–1170. 19941878 10.1016/j.neuropsychologia.2009.11.013 PMC2828502

[B6] Bartzokis G. Sultzer D. Lu P. H. Nuechterlein K. H. Mintz J. Cummings J. L. (2004). Heterogeneous age-related breakdown of white matter structural integrity: implications for cortical disconnection in aging and Alzheimer’s disease. *Neurobiol. Aging* 25 843–851. 10.1016/j.neurobiolaging.2003.09.005 15212838

[B7] Bugos J. A. Perlstein W. M. McCare C. S. Brophy T. S. Bedenbaugh P. H. (2007). Individualized piano instruction enhances executive functioning and working memory in older adults. *Aging Ment. Health* 11 464–471. 10.1080/13607860601086504 17612811

[B8] Caçola P. Roberson J. Gabbard C. (2013). Aging in movement representations for sequential finger movements: a comparison between young-, middle-aged, and older adults. *Brain Cogn* 82 1–5. 10.1016/j.bandc.2013.02.003 23501698

[B9] Carson R. G. Goodman D. Kelso J. A. S. Elliott D. (1995). Phase-transitions and critical fluctuations in rhythmic coordination of ipsilateral hand and foot. *J. Mot. Behav.* 27 211–224. 10.1080/00222895.1995.9941711 12529233

[B10] Caselli L. Iaboli L. Nichelli P. (2009). Time estimation in mild Alzheimer’s disease patients. *Behav. Brain Funct.* 5:32 10.1186/1744-9081-5-32 PMC2722666 19638203

[B11] Chen J. L. Zatorre R. J. Penhune V. B. (2006). Interactions between auditory and dorsal premotor cortex during synchronization to musical rhythms. *Neuroimage* 32 1771–1781. 10.1016/j.neuroimage.2006.04.207 16777432

[B12] Chu H. Yang C. Lin Y. Ou K. Lee T. O’Brien A. P. (2014). The impact of group music therapy on depression and cognition in elderly persons with dementia: a randomized controlled study. *Biol. Res. Nurs.* 16 209–217. 10.1177/1099800413485410 23639952

[B13] Clair A. A. C. O’Konski M. (2006). The effect of rhythmic auditory stimulation (RAS) on gait characteristics of cadence, velocity, and stride length in persons with late stage dementia. *J. Music Ther.* 43 154–163. 10.1093/jmt/43.2.154 16897907

[B14] Fling B. W. Seidler R. D. (2012). Fundamental differences in callosal structure, neurophysiologic function, and bimanual control in young and older adults. *Cereb. Cortex* 22 2643–2652. 10.1093/cercor/bhr349 22166764 PMC3464417

[B15] Fujiyama H. Garry M. I. Levin O. Swinnen S. P. Summers J. J. (2009). Age-related differences in inhibitory processes during interlimb coordination. *Brain Res.* 1262 38–47. 10.1016/j.brainres.2009.01.023 19368842

[B16] Fujiyama H. Garry M. I. Martin F. H. Summers J. J. (2010). An ERP study of age-related differences in the central cost of interlimb coordination. *Psychophysiology* 47 501–511. 10.1111/j.1469-8986.2009.00954.x 20102535

[B17] Fujiyama H. Hinder M. R. Garry M. I. Summers J. J. (2013). Slow and steady is not as easy as it sounds: interlimb coordination at slow speed is associated with elevated attentional demand especially in older adults. *Exp. Brain Res.* 227 289–300. 10.1007/s00221-013-3511-7 23591690

[B18] Giebel C. M. Sutcliffe C. Stolt M. Karlson S. Renom-Guiteras A. Soto M. (2014). Deterioration of basic activities of daily living and their impact on quality of life across different cognitive stages of dementia: a European study. *Int. Psychogeriatr.* 26 1283–1293. 10.1017/S1041610214000775 24784234

[B19] Halliday D. W. R. Stawski R. S. MacDonald S. W. S. (2016). Cognitively-impaired-not-demented status moderates the time-varying association between finger tapping inconsistency and executive performance. *Arch. Clin. Neuropsychol.* 2016 1–7. 10.1093/arclin/acw084 PMC5860477 27737850

[B20] Han S. A. (2016). Song-induced autobiographical memory of patients with early Alzheimer’s Dementia. *J. Music Hum. Behav.* 13 49–66. 10.21187/jmhb.2016.13.2.49

[B21] Han S. J. (2017). The relationship between self-perceived benefits from singing and quality of life in older adults aged over 75 years. *J. Music Hum. Behav.* 14 63–84. 10.21187/jmhb.2016.14.1.063

[B22] Hanna-Pladdy B. Gajewski B. (2012). Recent and past musical activity predicts cognitive aging variability: direct comparison with general lifestyle activities. *Front. Hum. Neurosci.* 6:198 10.3389/fnhum.2012.00198 PMC3400047 22833722

[B23] Haslinger B. Erhard P. Altenmüller E. Hennenlotter A. Schwaiger M. von Einsiedel H. G. (2004). Reduced recruitment of motor association areas during bimanual coordination in concert pianists. *Hum. Brain Mapp.* 22 206–215. 10.1002/hbm.20028 15195287 PMC6871883

[B24] Head D. Buckner R. L. Shimony J. S. Williams L. E. Akbudak E. Conturo T. E. (2004). Differential vulnerability of anterior white matter in nondemented aging with minimal acceleration in dementia of the Alzheimer type: evidence from diffusion tensor imaging. *Cereb. Cortex* 14 410–423. 10.1093/cercor/bhh003 15028645

[B25] Jahanshahi M. Saleem T. T. Ho A. K. Fuller R. R. Dirnberger G. (2009). A preliminary investigation of the running digit span as a test of working memory. *Behav. Neurol.* 20 17–25. 10.3233/BEN-2008-0212 PMC5452479 19491471

[B26] Kang Y. W. Chin J. H. Na D. L. (2002). A normative study of the digit span test for the elderly. *Korean J. Clin. Psychol.* 21 911–922.

[B27] Kaplan E. Fein D. Morris R. Delis D. C. (1991). *Wechsler Adult Intelligence Scale-Revised as a Neuropsychological Instrument.* San Antonio, TX: The Psychological Corporation.

[B28] Kauranen K. Vanharanta H. (1996). Influence of aging, gender, and handedness on motor performance of upper and lower extremities. *Percept. Mot. Skills* 82 515–525. 8724924 10.2466/pms.1996.82.2.515

[B29] Kilbreath S. L. Heard R. C. (2005). Frequency of hand use in healthy older persons. *Aust. J. Physiother.* 51 119–122. 10.1016/S0004-9514(05)70040-4 15924514

[B30] Krampe R. T. Doumas M. Lavrysen A. Rapp M. (2010). The costs of taking it slowly: fast and slow movement timing in older age. *Psychol. Aging* 25 980–990. 10.1037/a0020090 21186918

[B31] LaGasse A. B. Knight A. (2011). Rhythm and music in rehabilitation: a critical review of current research. *Crit. Rev. Phys. Rehabil. Med.* 23 49–67. 10.1615/CritRevPhysRehabilMed.v23.i1-4.40

[B32] Lipsitz L. A. (2004). Physiological complexity, aging, and the path to frailty. *Sci. Aging Knowledge Environ.* 2004:pe16 10.1126/sageke.2004.16.pe16 15103055

[B33] Mattay V. S. Fera F. Tessitore A. Hariri A. R. Das S. Callicott J. H. (2002). Neurophysiological correlates of age-related changes in human motor function. *Neurol* 58 630–635. 10.1212/WNL.58.4.630 11865144

[B34] McAuley J. D. Jones M. R. Holub S. Johnston H. M. Miller N. S. (2006). The time of our lives: life span development of timing and event tracking. *J. Exp. Psychol.* 135 348–367. 10.1037/0096-3445.135.3.348 16846269

[B35] Meesen R. L. J. Wenderoth N. Temprado J. Summers J. J. Swinnen S. P. (2006). The coalition of constraints during coordination of the ipsilateral and heterolateral limbs. *Exp. Brain Res.* 174 367–375. 10.1007/s00221-006-0471.1 16819649

[B36] Nagahama Y. Okina T. Suzuki N. (2015). Impaired imitation of gestures in mild dementia: comparison of dementia with Lewy bodies, Alzheimer’s disease and vascular dementia. *J. Neurol. Neurosurg. Psychiatry* 86 1248–1252. 10.1136/jnnp-2014-309436 25515503

[B37] Park D. C. (2000). “The basic mechanisms accounting for age-related decline in cognitive function,” in *Aging and Cognition: A Primer* eds Park D. C. Schwarz N. (New York, NY: Psychology Press) 3–22.

[B38] Pecenka N. Engel A. Keller P. E. (2013). Neural correlates of auditory temporal predictions during sensorimotor synchronization. *Front. Hum. Neurosci.* 7:380 10.3389/fnhum.2013.00380 PMC3748321 23970857

[B39] Reitan R. M. (1956). *Trail Making Test: Manual for Administration, Scoring, and Interpretation.* Indianapolis, IN: Indiana University.

[B40] Ridderikhoff A. Peper C. L. E. Beek P. J. (2008). Attentional loads associated with interlimb interactions underlying rhythmic bimanual coordination. *Cognition* 109 372–388. 10.1016/j.cognition.2008.10.002 19014874

[B41] Schlaug G. (2001). The brain of musicians. *Ann. N. Y. Acad. Sci.* 930 281–299. 10.1111/j.1749-6632.2001.tb05739.x 11458836

[B42] Seidler R. D. Bernard J. A. Burutolu T. B. Fling B. W. Gordon M. T. Gwin J. T. (2010). Motor control and aging: links to age-related brain structural, functional, and biochemical effects. *Neurosci. Biobehav. Rev.* 34 721–733. 10.1016/j.neubiorev.2009.10.005 19850077 PMC2838968

[B43] Serrien D. J. Swinnen S. P. Stelmach G. E. (2000). Age-related deterioration of coordinated interlimb behavior. *J. Gerontol. B Psychol. Sci. Soc. Sci.* 55 295–303. 10.1093/geronb/55.5.P295 10985294

[B44] Shetty A. K. Shanker V. Annamalai N. (2014). Bimanual coordination: influence of age and gender. *J. Clin. Diagn. Res.* 8 15–16. 10.7860/JCDR/2014/7333.3994 24701469 PMC3972539

[B45] Sullivan E. V. Pfefferbaum A. (2002). Diffusion tensor imaging and aging. *Neurosci. Biobehav. Rev.* 30 749–761. 10.1016/j.neubiorev.2006.06.002 16887187

[B46] Temprado J. J. Zanone P. G. Monno A. Laurent M. (2001). A dynamical framework to understand performance trade-offs and interference in dual tasks. *J. Exp. Psychol.* 27 1303–1313. 10.1037/0096-1523.27.6.1303 11766926

[B47] Trapp S. Lepsien J. Sehm B. Villringer A. Ragert P. (2012). Changes of hand switching costs during bimanual sequential learning. *PLOS ONE* 7:e45857 10.1371/journal.pone.0045857 PMC3448681 23029279

[B48] Trombetti A. Hars M. Hermann F. R. Kressig R. W. Ferrari S. Rizzoli R. (2011). Effect of music-based multitask training on gait, balance, and fall risk in elderly people: a randomized controlled trial. *Arch. Intern. Med.* 171 525–533. 10.1001/archinternmed.2010.446 21098340

[B49] Trugeon M. Wing A. M. Taylor L. W. (2011). Timing and aging: slowing of fastest regular tapping rate with preserved timing error detection and correction. *Psychol. Aging* 26 150–161. 10.1037/a0020606 20973598

[B50] Tucker M. G. Kavanagh J. J. Barrett R. S. Morrison S. (2008). Age-related differences in postural reaction time and coordination during voluntary sway movements. *Hum. Mov. Sci.* 27 728–737. 10.1016/j.humov.2008.03.002 18513814

[B51] Vaillancourt D. E. Newell K. M. (2002). Changing complexity in human behavior and physiology through aging and disease. *Neurobiol. Aging* 23 1–11. 10.1016/S0197-4580(01)00247-0 11755010

[B52] van der Wardt V. Hood L. P. Booth V. Masud T. Harwood R. (2015). The association of specific executive functions and fall risk in people with mild cognitive impairment and early-stage dementia. *Dement Geriatr. Cogn. Disord.* 40 178–185. 10.1159/000433523 26206201

[B53] Vanneste S. Pouthas V. Wearden J. H. (2001). Temporal control of rhythmic performance: a comparison between young and old adults. *Exp. Aging Res.* 27 83–102. 10.1080/03610730125798 11205531

[B54] Voelcker-Rehage C. (2008). Motor-skill learning in older adults: a review of studies on age-related differences. *Eur. Rev. Aging Phys. Act.* 5 5–16. 10.1007/s11556-008-0030-9

[B55] Voelcker-Rehage C. Godde B. Staudinger U. M. (2011). Cardiovascular and coordination training differentially improve cognitive performance and neural processing in older adults. *Front. Hum. Neurosci.* 5:26 10.3389/fnhum.2011.00026 PMC3062100 21441997

[B56] Witt S. T. Laird A. R. Meyerand M. E. (2008). Functional neuroimaging correlates of finger-tapping task variations: an ALE meta-analysis. *Neuroimage* 42 343–356. 10.1016/j.neuroimage.2008.04.025 18511305 PMC2592684

[B57] Yi H. Chin J. Lee B. H. Kang Y. Na D. L. (2007). Development and validation of Korean version of trail making test for elderly persons. *Dement Neurocogn. Disord.* 6 54–66.

